# The impact of e-cigarette use on cognitive function, emotional intelligence, and dementia risk in adolescents and young adults

**DOI:** 10.1038/s41598-026-48579-z

**Published:** 2026-04-12

**Authors:** Sasikan Chaleechad, Thananya Nentakong, Phiangphim Punrasi, Aroon La-up, Anupon Tadee, Theerayut Baubhom

**Affiliations:** 1 Community Public Health Program, Mahidol University, Nakhonsawan Campus, Nakhonsawan, 60130 Thailand; 2 School of Nursing, Mahidol University, Nakhonsawan Campus, Nakhonsawan, 60130 Thailand; 3 Department of Environmental Health Sciences, School of Public Health, University of Michigan, Ann Arbor, MI 48109 USA

**Keywords:** E-cigarette use, Adolescents, ADHD symptoms, Nicotine exposure, Mental health, Young smokers, Diseases, Health care, Medical research, Neurology, Neuroscience, Risk factors

## Abstract

The increasing prevalence of e-cigarette use among adolescents and young adults has raised concerns about its potential association with cognitive and behavioral functions. While nicotine exposure is known to affect brain development, its associations with Attention-Deficit/Hyperactivity Disorder (ADHD) symptoms, emotional intelligence (EQ), and dementia risk remain unclear. This study primarily examined the association between e-cigarette use and cognitive impairment assessed by the Montreal Cognitive Assessment (MoCA). Secondary analyses explored associations with ADHD symptom tendencies and emotional intelligence. A cross-sectional analytical study was conducted in Lat Krabang District, Bangkok, Thailand, with 232 participants aged 18–25 years, equally divided into e-cigarette users (n = 116) and non-smokers (n = 116). Data were collected using the Adult ADHD Self-Report Scale (ASRS-v1.1, 18 items), the Emotional Quotient Test, and the Montreal Cognitive Assessment (MoCA). Statistical analyses compared cognitive function, EQ levels, and dementia risk between the two groups. No significant differences were found in ADHD symptom tendencies and EQ levels between e-cigarette users and non-smokers. However, e-cigarette users exhibited a significantly higher proportion of dementia risk compared to non-smokers. Notably, individuals with no intention to quit within one month had a sixfold increased dementia risk, while those without plans to quit within six months had a fourfold increased risk. No other personal factors significantly influenced dementia risk. While e-cigarette use was not significantly associated with ADHD symptom tendencies or EQ levels, it was associated with a higher likelihood of dementia risk. These findings suggest potential neurocognitive concerns related to e-cigarette use; however, causal inferences cannot be made due to the cross-sectional design. Further longitudinal studies are warranted to clarify these relationships.

**Supplementary Information:**

The online version contains supplementary material available at 10.1038/s41598-026-48579-z.

## Introduction

E-cigarettes, or electronic nicotine delivery systems (ENDS), are battery-powered devices that heat a liquid containing nicotine and various chemicals, creating an aerosol for users to inhale. Available in multiple forms, including disposable e-cigarettes (Cigalike), refillable models (Pen-style, Tank), and more advanced variants such as Mods and Pod Systems, e-cigarettes have gained significant popularity, especially among adolescents and young adults. A newer variant, IQOS, is classified as a heated tobacco product (HTP), which differs from e-cigarettes (ENDS) as it heats tobacco rather than liquid nicotine solutions, aiming to reduce harmful chemical exposure^1^. The increasing variety and accessibility of e-cigarettes have contributed to their widespread use, particularly among younger populations. Despite being marketed as a less harmful alternative to traditional cigarettes, e-cigarettes still contain harmful substances, including nicotine, propylene glycol, glycerin, and flavoring agents, all of which pose serious health risks^2^. Nicotine exposure during adolescence can interfere with brain development, impair cognitive functions, and heighten addiction susceptibility. Many Thai adolescents perceive e-cigarettes as safer than traditional cigarettes, which contributes to increased usage, often driven by factors such as cost, reusability, and misconceptions about smoking cessation^1^. However, excessive use has been associated with acute respiratory issues, such as airway constriction and pneumonia^3^.

Thailand has strict laws prohibiting the sale, import, and possession of e-cigarettes under the Ministry of Commerce’s 2014 ban and the Consumer Protection Board’s 2015 order. However, enforcement remains challenging due to their widespread availability through online platforms. This misconception is further reinforced by misleading advertising and social media campaigns, which contribute to the normalization of e-cigarette use^2^.

Although e-cigarettes are designed to reduce nicotine intake compared to traditional cigarettes, their aerosol still contains harmful chemicals. Long-term exposure has been linked to neurological effects, sleep disturbances, dizziness, and increased risks of cardiovascular diseases, gastrointestinal problems, and cancer^4^. Emerging evidence suggests a link between e-cigarette use and cognitive or behavioral changes, particularly in areas such as impulse control and attention (need citations? ). However, limited research has examined the direct association between e-cigarette use and Attention-Deficit/Hyperactivity Disorder (ADHD) symptoms.

ADHD, a neurodevelopmental disorder, is characterized by deficits in executive functions, including attention, impulse control, and decision-making. Diagnosed primarily in childhood, ADHD persists into adulthood in many individuals. It is recognized under both the Diagnostic and Statistical Manual of Mental Disorders, 5th Edition, Text Revision (DSM-5-TR), and the International Classification of Diseases (ICD-10) as Hyperkinetic Disorder. The disorder is marked by three core symptoms: inattention (difficulty sustaining focus), hyperactivity (excessive movement), and impulsivity (acting without thinking, interrupting conversations)^5^. Nicotine, a powerful neurotoxin, can influence neurotransmitter regulation, particularly acetylcholine, dopamine, and norepinephrine, key neurotransmitters responsible for regulating cognitive functions. Adolescents, whose brains are still developing, are particularly vulnerable to nicotine addiction, which may exacerbate ADHD symptoms^6^.

Nicotine acts directly on the brain’s neurochemical pathways, leading to chronic stimulation of neural circuits and contributing to addiction. Although e-cigarettes are often perceived as a safer alternative to conventional smoking, accumulating evidence indicates that their aerosol still contains harmful substances, including nicotine, toxic metals, and other chemical compounds^7^. Neurotoxicological research suggests that exposure to these substances may disrupt calcium homeostasis, impair autophagy, and induce mitochondrial dysfunction, leading to neuroinflammation and oxidative stress. These mechanisms can interfere with the prefrontal cortex, a brain region critical for attention regulation, decision-making, and emotional control, and may therefore be associated with changes in cognitive and behavioral functions, including ADHD-related symptoms^8^.

In addition to cognitive and behavioral factors, emotional intelligence (EQ) plays a crucial role in regulating human cognition and behavior. Emotional intelligence, primarily associated with the functioning of the right hemisphere of the brain, involves the ability to perceive, assess, and manage one’s own emotions and those of others. Key components of emotional intelligence include good (Dii), the ability to regulate emotions and demonstrate empathy and social responsibility; competence (Keng), the ability to understand oneself, make decisions, and build relationships; and well-being (Suk), the ability to achieve contentment and inner peace^9^. Daniel Goleman identified five core components of emotional intelligence: self-awareness, self-regulation, self-motivation, recognizing emotions in others, and social skills^10^.

Furthermore, nicotine exposure during pregnancy has been linked to impaired fetal brain development, raising the risk of cognitive and behavioral disorders, including ADHD. Prenatal nicotine exposure can lead to long-term consequences through structural and functional changes in brain regions that regulate attention, decision-making, and emotional control^7^. Given the rising prevalence of e-cigarette use among adolescents and its potential association with cognitive function, this study aims to explore the relationship between e-cigarette use and ADHD tendencies in young adults aged 18–25 years. This research seeks to provide empirical evidence on how nicotine exposure through e-cigarettes affects ADHD-related symptoms, contributing to public health policy and intervention strategies.

Dementia is characterized by progressive deterioration in cognitive functions such as memory, reasoning, and judgment. Cognitive impairment may be influenced by neurotoxic exposures, including nicotine, which has been shown to affect brain structure and function. Understanding early indicators of cognitive decline in young populations may provide insight into long-term neurological risks^11^.

These three domains—ADHD symptoms, emotional intelligence, and cognitive function—represent interconnected aspects of neurocognitive and psychosocial functioning. Nicotine exposure has been associated with attention regulation (ADHD-related symptoms), emotional regulation and interpersonal functioning (EQ), and broader cognitive performance (as assessed by MoCA). These domains may reflect shared neurocognitive pathways, particularly involving executive function, emotional regulation, and reward processing, which are known to be influenced by nicotine exposure. Therefore, examining these outcomes together allows for a more integrated assessment of neurobehavioral functioning.

Therefore, this study primarily examined the association between e-cigarette use and cognitive impairment assessed using the Montreal Cognitive Assessment (MoCA). Secondary analyses explored potential associations with ADHD symptom tendencies and EQ among young adults. By exploring these connections, the research seeks to provide comprehensive insights into the cognitive, emotional, and behavioral effects of e-cigarette use, contributing to the development of more effective public health policies and intervention strategies.

## Materials and methods

### Study design

This cross-sectional analytical study was conducted from August 2023 to July 2024 to examine the associations between e-cigarette use, ADHD symptom tendency, EQ, and dementia risk among young adults aged 18–25 years. Data were collected through structured questionnaires, standardized ADHD assessments, EQ evaluations, and questions related to e-cigarette use patterns. The research protocol was approved by the Human Subjects Research Committee of the Mahidol University Central Institutional Review Board (MU-CIRB) prior to the commencement of the study. Informed consent was obtained from all participants, and confidentiality was ensured throughout the study.

### Study area

This research was conducted in Lat Krabang District, Bangkok, Thailand, an urbanized area located on the eastern outskirts of Bangkok. Lat Krabang is one of the fifty districts of Bangkok, covering an area of approximately 123.859 square kilometers covering 64,228 family houses in 63 communities, 1 university and 20 schools^12,13^. The district is characterized by a mix of residential, industrial, and educational zones, with a growing population due to urban expansion and economic development^14^.

### Participants

The study included individuals aged 18–25 years, who self-identified their gender/sexual identity as male, female, or LGBTQ+, residing in Lat Krabang District, Bangkok, Thailand. Participants were asked to self-identify their gender/sexual identity using these three categories. It should be noted that this variable reflects self-reported gender/sexual identity and does not distinguish between sex assigned at birth and gender identity or sexual orientation, and therefore should be interpreted with caution. The objective was to assess the association between electronic cigarette (e-cigarette) use and neurocognitive and psychosocial outcomes by comparing e-cigarette users with non-smokers.

The sample size was determined based on the study by Jason R. and Joseph S. B^15^., which reported an odds ratio (OR) of 0.510 and a P-value < 0.001 for vaping behavior. Using G*Power Version 3.1.9.4, the sample size calculation was based on a two-tailed test with OR = 0.5, α = 0.05, power = 0.95, and probability = 1. The initial sample size was 210 participants, increased by 10% to 231 to account for potential data loss. To maintain equal group sizes, the final total was rounded to 232 participants (n), with 116 e-cigarette users and 116 non-smokers.

As of November 2023, the total population of individuals aged 18–25 years in Lat Krabang District was 18,547 (N), with 9,099 males (49.06%) and 9,448 females (50.94%), based on the Health Promotion and Environmental Health Information System. A systematic random sampling method was applied, with the sampling interval (I) calculated as I = N/n, where N is the total number of eligible individuals and n is the required sample size. A random number between 1 and 9 was selected as the starting point. Participants were recruited in sequence upon entering the study area. If a selected individual declined or had already participated, the next eligible person was recruited. The e-cigarette user group was completed first, followed by the control group. Each participant received a unique identification number for record-keeping.

Inclusion criteria required participants to be aged 18–25 years, with e-cigarette users having vaped for over a year and still using e-cigarettes, while non-smokers were defined as individuals who had never used any nicotine-containing products, including e-cigarettes, conventional cigarettes, or other tobacco products. Exclusion criteria included individuals with psychiatric disorders or undergoing psychiatric treatment, those with disabilities requiring full-time assistance, individuals unable to communicate in Thai, traditional cigarette users or dual users, former e-cigarette users who had recently quit, and those unwilling to participate.

### Instruments

The research utilized a comprehensive questionnaire (shown in Supplementary Material file), divided into six sections, to assess various aspects of the participants’ behaviors and cognitive functions. Sections 1 to 3, covering demographic information, smoking and substance use behaviors, and e-cigarette usage, were validated by three experts, yielding a Content Validity Index (CVI) average of 0.79 and a Cronbach’s alpha reliability coefficient of 0.891, demonstrating strong internal consistency. Section 4 employed the Adult ADHD Self-Report Scale (ASRS) Version 1.1, a tool developed by Kessler et al. to screen for ADHD symptom tendency based on DSM-5 criteria^16^. In this study, the term “ADHD symptom tendency” refers to screening results obtained from the ASRS and does not represent a clinical diagnosis of ADHD. Section 5 assessed EQ using a 52-item Likert-scale format, designed to evaluate emotional awareness, self-regulation, empathy, and social skills, as outlined by the Ministry of Public Health^9^. EQ was evaluated using a five-point Likert scale, with results categorized into three levels based on the classification method: above-normal (3.01–4.00), normal (2.01–3.00), and below-normal (1.00–2.00)^17^. The final section utilized the Montreal Cognitive Assessment (MoCA) to assist in the early detection of dementia, evaluating cognitive functions such as attention, memory, and executive function^18,19^. Each section’s relevance and alignment with the research objectives were further assessed using the Item-Objective Congruence (IOC) index, with values ranging from 0.50 to 0.79, ensuring the questionnaire’s validity for data collection.

### Procedures

The data collection process followed a systematic procedure. First, the researcher obtained an official authorization letter from Mahidol University and submitted it to the Director of Lat Krabang District Office to request permission for data collection. This step ensured compliance with administrative procedures and facilitated coordination with local authorities. Next, the researcher conducted fieldwork in Lat Krabang District, Bangkok, selecting high-traffic public areas such as university surroundings, coffee shops, shopping malls, restaurants, and transit stations for participant recruitment. These locations were chosen to maximize the likelihood of encountering individuals within the study’s target age group. Before data collection, the researcher explained the study objectives, potential benefits, and confidentiality measures to participants. Volunteers were informed that all collected data would remain confidential and be destroyed after the study’s completion. Participants were then asked to provide written consent by signing the informed consent form before proceeding with the study.

The researcher provided clear instructions on how to complete the questionnaire, ensuring participants understood each section. Participants were then given approximately 35 to 40 min to complete the questionnaire independently. Finally, the researcher reviewed the completed questionnaires for accuracy and completeness before proceeding with data analysis. This step ensured that all collected data met the required standards for further processing and interpretation.

### Statistical analysis

Data from the questionnaires were analyzed using inferential statistics to assess the association between e-cigarette use and the likelihood of developing ADHD symptoms, EQ levels, and the risk of dementia. The independent variables included the number of cigarettes smoked per day, the duration of smoking, e-cigarette usage behaviors, and the trend of cigarette use. Confounding variables included data on tobacco, alcohol, and substance use. The dependent variables were ADHD symptom tendencies, EQ levels, and dementia risk. Pearson’s Chi-square test was used to examine differences in the prevalence of ADHD symptom tendency, EQ levels, and dementia risk between e-cigarette users and non-smokers. To control for potential confounding variables, binary logistic regression analysis was performed to estimate adjusted odds ratios (ORs) and 95% confidence intervals (CIs) for factors associated with dementia risk. A confidence level of 95% was applied, and statistical significance was set at a *P*-value of less than 0.05. All analyses were conducted using IBM SPSS Statistics version 29 (Armonk, NY). Descriptive statistics were used to summarize participant characteristics, substance use history, and e-cigarette usage behaviors. Categorical variables were presented as frequencies and percentages, while continuous variables were reported as means, medians, minimum (min), and maximum (max) values.

## Results

### Demographic analysis results for all participants

The study included 232 adolescents aged 18–25 years residing in Lat Krabang District, Bangkok. Among them, 53.9% were female, 40.9% were male, and 5.2% self-identified as LGBTQ+ under the combined gender/sexual identity category. The mean age was 22 years (SD = 1.97), with the youngest at 18 years (7.8%) and the oldest at 25 years (21.1%). The mean BMI was 23.19, with 38.8% in the normal range (18–23), 20.7% underweight (BMI < 18), 12.5% overweight (BMI > 23), and 14.2% classified as obese (BMI > 30). Regarding education, 50.9% held a bachelor’s degree or higher, followed by 33.6% with high school or vocational education. Most participants were single (94.0%), with 87.1% having no children. The majority lived in households of 4–6 members (61.6%) and 60.3% resided in private homes, while others lived in dormitories or rented accommodations. Most had no chronic illness (90.5%), and 58.6% were students, followed by 29.3% working in private companies. The most common income range was 5,001–10,000 THB (~ 148.3–296.55 USD) per month (39.2%), while 37.1% earned above 10,000 THB (~ 296.55 USD) and 23.7% earned below 5,000 THB (~ 148.28 USD). All details shown in Table 1.

**Table 1:** Descriptive statistics of general characteristics of participants (*n* = 232).

**Variables**	**Non-smoker ** **(%) ** **(n= 116)**	**E-cigarette user (%) ** **(n= 116)**	**All participants (%) ** **(n = 232)**
Self-reported gender/sexual identity			
Male	35 (30.2)	60 (51.7)	95 (40.9)
Female	73 (62.9)	52 (44.8)	125 (53.9)
LGBTQ+	8 (6.9)	4 (3.4)	12 (5.2)
Age (years)			
18	6 (5.2)	12 (10.3)	18 (7.8)
19	1 (0.9)	2 (1.7)	3 (1.3)
20	1 (0.9)	10 (8.6)	11 (4.7)
21	23 (19.8)	18 (15.5)	41 (17.7)
22	51 (44.0)	20 (17.2)	71 (30.6)
23	12 (10.3)	18 (15.5)	30 (12.9)
24	3 (2.6)	6 (5.2)	9 (3.9)
25	19 (16.4)	30 (25.9)	49 (21.1)
(Mean = 22.22, S.D = 1.969, Median = 22.0, Min = 18, Max = 25)			
BMI			
Underweight (< 18.5)	19 (16.4)	29 (25.0)	40 (20.7)
Normal weight (18.6-22.9)	48 (41.4)	42 (36.2)	90 (38.8)
Overweight (≥ 23)	16 (13.8)	13 (11.2)	29 (12.5)
Obese (≥ 30)	33 (28.5)	32 (27.6)	65 (28.0)
(Mean = 23.19, S.D = 5.73, Median = 21.82, Min = 14.36, Max = 45.37)			
Education level			
Did not attend school	0 (0)	2 (1.7)	2 (0.9)
Primary school	0 (0)	4 (3.4)	4 (1.7)
High school/Vocational	26 (22.4)	52 (44.8)	78 (33.6)
Diploma/High certificate	16 (13.8)	14 (12.1)	30 (12.9)
Bachelor’s degree or higher	74 (63.8)	44 (37.9)	118 (50.9)
Marital status			
Single	112 (96.6)	106 (91.7)	218 (94.0)
Married	4 (3.4)	6 (5.2)	10 (4.3)
Separated/divorced	0 (0)	4 (3.4)	4 (1.7)
Household members			
1-3 members	24 (20.7)	35 (30.2)	59 (25.4)
4-6 members	81 (69.8)	62 (53.4)	143 (61.6)
> 6 members	11 (9.5)	19 (16.4)	30 (12.9)
Residence type			
Private home	62 (53.4)	78 (67.2)	140 (60.3)
Dormitory (on-campus)	26 (22.4)	18 (15.5)	44 (19.0)
Dormitory (off-campus)	17 (14.7)	12 (10.3)	29 (12.5)
Others	11 (9.5)	8 (6.9)	19 (8.2)
Personal diseases			
No	100 (86.2)	110 (94.8)	210 (90.5)
Yes	16 (13.8)	6 (5.2)	22 (9.5)
Occupations			
Student	82 (70.7)	54 (46.6)	136 (58.6)
Private business	2 (1.7)	8 (6.9)	10 (4.3)
Government employee	2 (1.7)	0 (0)	2 (0.9)
Non-government employee	24 (20.7)	44 (37.9)	68 (29.3)
Unemployed	2 (1.7)	4 (3.4)	6 (2.6)
Others	4 (3.4)	6 (5.2)	10 (4.3)
Monthly income (THB/Month)			
0-5,000	42 (36.2)	13 (11.2)	55 (23.7)
5,001-10,000	40 (34.5)	51 (44.0)	91 (39.2)
> 10,000	34 (29.3)	52 (44.8)	86 (37.1)

Gender/sexual identity was self-reported. The category “LGBTQ+” may include diverse gender identities and/or sexual orientations. This variable does not distinguish between sex assigned at birth, gender identity, and sexual orientation. Body Mass Index. BMI categories were classified as underweight (<18.5 kg/m²), normal (18.5–22.9 kg/m²), overweight (≥23 kg/m²), and obese (≥30 kg/m²). Values are presented as frequency (%).

The study revealed that among the 232 participants, 72.8% reported consuming alcoholic beverages, while 23.7% did not consume alcohol, and 3.4% had previously consumed but had since quit. Regarding substance use, the majority (95.3%) reported never using drugs, whereas 3.9% had a history of drug use, and 0.9% had used drugs in the past but had since quit. Additionally, nearly half of the participants (49.1%) had at least one family member who smoked, while 50.9% reported having no smokers in their household. These findings highlight the prevalence of alcohol consumption among young adults and the relatively low rates of substance use in the study population. All data presented in Supplementary Table S1.

The study examined the behavior of 116 adolescents aged 18–25 years who use e-cigarettes, comparing them with a non-smoking group, all results shown in Supplementary Table S2. The findings revealed that most e-cigarette users (81.9%) smoked one or fewer devices per day, while 18.1% smoked more than one device per day. Regarding the age of first use, the most of users (33.6%) began smoking between 17 and 19 years old, followed by 23.3% who started at ages 14–16. The primary reasons for using e-cigarettes were curiosity and the desire to try smoking (16.8%), followed by preferences for taste or scent (13.4%), and the belief that e-cigarettes are less harmful than traditional cigarettes (10.7%). Additionally, 10% of users reported smoking due to the convenience of use, while other factors such as stress, peer influence, or social settings also played a role. Most e-cigarette use occurred between 8:01 PM and midnight (25.3%), followed by 20.9% of users smoking between 4:01 PM and 8:00 PM. In terms of expenditure, the majority (42.2%) spent between 100 and 500 THB per month on e-cigarettes, while 26.7% spent less than 100 THB, and 21.6% spent between 500 and 1,000 THB per month. These findings highlight the significant influence of curiosity, peer pressure, and the perceived reduced harm of e-cigarettes as key factors driving usage among young adults.

The study on e-cigarette use among adolescents aged 18–25 years revealed detailed behavioral patterns among 116 users, presented in Supplementary Table S3. Most participants (36.2%) used e-cigarettes 2–5 times per day, followed by 33.6% who used them more than 10 times per day. Most adolescents reported using e-cigarettes while drinking alcohol (26.8%) or at social gatherings (18%). A significant proportion (33.6%) did not have a specific time for use, while others smoked after meals or during work/school hours. In terms of location, 33.6% did not specify a particular place, while 31% used e-cigarettes in entertainment venues, and 28.4% at home or dormitories. Most participants purchased e-cigarettes from general stores (33.3%) or online (30.2%), with a large proportion discovering e-cigarette ads on social media (36.2%). Regarding the future of e-cigarette use, half of the participants showed a tendency to decrease usage, while 40.5% expressed a desire to quit within one month, and 40.2% wanted to quit within six months. Despite these intentions, 44% had never attempted to quit, and 31% had made attempts but relapsed. These findings suggest that while a significant number of adolescents wish to quit or reduce e-cigarette use, there is a high rate of ongoing usage, with social environments and easy access being influential factors.

### Association between e-cigarette use and ADHD symptom prevalence

The data, as presented in Supplementary Table S4, show that the most participants (89.2%) did not exhibit symptoms consistent with ADHD, while 10.8% of participants showed symptoms indicative of the disorder. Specifically, among the 207 participants without ADHD symptoms, most were in the non-smoking group. In contrast, a slightly higher proportion of participants with ADHD-like symptoms were found in the e-cigarette user group. This table provides a clearer understanding of how ADHD symptoms are distributed across the different groups, highlighting the low overall incidence of ADHD in the sample and offering insight into the potential relationship between smoking behavior and ADHD symptoms.

The analysis of the association between e-cigarette use and ADHD symptom tendency was conducted using the Pearson Chi-Square Test. According to the data presented in Table 2, thirteen (11.2%) participants in the e-cigarette user group exhibited ADHD symptom tendency, compared to 12 (10.3%) participants among non-smokers. The statistical analysis revealed no significant difference in ADHD symptom tendency between e-cigarette users and non-smokers at a significance level of 0.05. These findings suggest that e-cigarette use was not significantly associated with ADHD symptom tendency in the studied population.

**Table 2:** Chi-square analysis of the association between ADHD symptom tendency and smoking behavior in e-cigarette users and non-smokers.

ADHD symptom prevalence	Non-smoker (*n* = 116) *n* (%)	E-cigarette user (*n* = 116) *n* (%)	Pearson Chi-Square	*p*-value
No symptoms consistent with ADHD	104 (89.7)	103 (88.8)	0.045	0.832
Symptoms consistent with ADHD	12 (10.3)	13 (11.2)		

ADHD symptom tendency was assessed using the ASRS. A score ≥ 24 indicates ADHD-consistent symptoms. Pearson’s Chi-square test was used to compare groups. The Pearson Chi-square test (χ² = 0.045, *p* = 0.832) showed no significant association between ADHD symptom prevalence and smoking behavior (p-value > 0.05).

### Emotional intelligence (EQ) levels among e-cigarette users and non-users

The overall EQ level of the participants (*n* = 232) was within the normal range; however, variations were observed across different EQ dimensions. In the “Good” category of EQ, which includes self-regulation, empathy, and responsibility, self-regulation was found to be within the normal range, while empathy and responsibility were below normal. Similarly, in the “Competent” category, which assesses motivation, decision-making, and relationship skills, motivation was at a normal level, whereas decision-making and relationship-building skills were below normal. Lastly, in the “Well-Being” category, which includes self-pride, self-satisfaction, and inner peace, self-pride remained within the normal range, while self-satisfaction and inner peace were below normal. All data of EQ analysis were presented in Supplementary Table S5. These findings suggest that while overall EQ levels were within the normal range, specific aspects related to empathy, decision-making, interpersonal relationships, and emotional well-being were lower than expected. The observed patterns indicate potential areas for intervention to improve emotional regulation and interpersonal skills, particularly among e-cigarette users. Further analysis is needed to explore the relationship between EQ levels and e-cigarette use behaviors to determine whether e-cigarette consumption plays a role in lower scores across these emotional dimensions.

EQ level assessment among non-smokers (*n* = 116), in the Supplementary Table S6 “Good” dimension of EQ, overall emotional intelligence levels were found to be within the normal range. However, when examining specific aspects, self-regulation was within the normal range, whereas empathy and responsibility were below the normal level. For the “Competent” dimension, the overall EQ level was below normal. When broken down into individual aspects, all evaluated components—including motivation, decision-making, and interpersonal relationships—were also below normal. Regarding the “Well-Being” dimension, the overall EQ level was below normal. Similarly, all aspects, including self-pride, self-satisfaction, and inner peace, were found to be below normal.

EQ level assessment among e-cigarette users (*n* = 116), in the Supplementary Table S7 presented to the “Good” dimension of EQ, the overall EQ level among e-cigarette users was within the normal range. However, when examining specific aspects, self-regulation remained at a normal level, whereas empathy and responsibility were below normal. In the “Competent” dimension, the overall EQ level was also within the normal range. When analyzing individual aspects, motivation was at a normal level, while decision-making and interpersonal relationships were below normal. Regarding the “Well-Being” dimension, the overall EQ level was within the normal range. However, when examined separately, self-pride was within the normal range, whereas self-satisfaction and inner peace were below normal.

The comparative findings suggest that while both groups exhibited weaknesses in empathy, responsibility, decision-making, interpersonal relationships, and emotional well-being, the deficits were more pronounced among non-smokers. Non-smokers demonstrated lower overall EQ levels in both the “Competent” and “Well-Being” dimensions, while e-cigarette users retained normal levels in these dimensions, except for specific aspects. Interestingly, a higher proportion of non-smokers exhibited below-normal scores in the “Competent” EQ dimension compared to e-cigarette users. However, this difference was not statistically significant and should be interpreted with caution. One possible explanation relates to the social context of vaping. E-cigarette use often occurs within peer-group environments, which may facilitate social interaction and relationship-building. In this study, a proportion of participants reported peer influence and socialization as reasons for initiating e-cigarette use. This interpretation is consistent with previous studies indicating that e-cigarette use among young adults is often driven by social motivations and peer-related factors, which are also associated with interpersonal functioning and social engagement. Additionally, the “Competent” dimension includes relationship-building skills, which may overlap conceptually with social sensitivity and peer interaction. Therefore, the observed pattern may reflect differences in social context rather than true differences in emotional intelligence. This may also reflect potential construct overlap between social sensitivity, peer influence, and the relationship-building component of the EQ measure. The observed patterns highlight potential areas for intervention, particularly in fostering empathy, improving decision-making skills, and enhancing emotional well-being. Further research is necessary to explore the causal relationship between e-cigarette use and EQ development, as well as to determine whether lifestyle factors, peer influences, or other external variables contribute to these differences.

Statistical analysis of the relationship between EQ and e-cigarette use, the relationship between the “Good” dimension of EQ and e-cigarette was analyzed using the Chi-square test to examine the association between e-cigarette use and the “Good” dimension of emotional intelligence, which includes self-regulation, empathy, and responsibility. Based on the proportions presented in Table 3, **28**.4% (*n* = 33) of e-cigarette users exhibited below-normal EQ levels in this dimension, compared to 19.8% (*n* = 23) of non-smokers. Despite this difference in proportions, the statistical analysis revealed no significant association between e-cigarette use and the “Good” dimension of EQ(p-value > 0.05).

The relationship between the “Competent” dimension of EQ and e-cigarette use found that the “Competent” dimension, which assesses motivation, decision-making, and interpersonal relationships, the analysis in Table 10 showed that 12.9% (*n* = 15) of non-smokers had below-normal levels in this dimension, compared to 6.0% (*n* = 7) of e-cigarette users. While non-smokers exhibited a higher proportion of below-normal EQ scores, the Chi-Square test indicated no statistically significant relationship between e-cigarette use and the “Competent” dimension of EQ at the 0.05 significance level (*p* > 0.05). Lastly, the relationship between the “Well-Being” dimension of EQ and e-cigarette use, the result for the “Well-Being” dimension, which includes self-pride, self-satisfaction, and inner peace, was also analyzed in relation to e-cigarette use. The results presented in Table 10 revealed that 9.5% (*n* = 11) of non-smokers had below-normal levels in this dimension, compared to 7.8% (*n* = 9) of e-cigarette users. The statistical test showed no significant association between e-cigarette use and the “Well-Being” dimension of EQ at the 0.05 significance level (*p* > 0.05). All findings from the EQ evaluation suggest that while there were differences in the proportion of individuals with below-normal EQ levels across the three dimensions, these differences were not statistically significant. This indicates that e-cigarette use may not be a key determinant of emotional intelligence levels in this sample. However, the observed trends suggest that non-smokers tended to have lower EQ scores in the “Competent” and “Well-Being” dimensions, while e-cigarette users showed a higher proportion of below-normal scores in the “Good” dimension. Further research with a larger sample size and additional confounding variables should be conducted to validate these findings.

**Table 3:** Analysis of the relationship between EQ dimensions (good, competent, and well-being) and e-cigarette use using the Chi-square test.

EQ levels	Non-smoker (*n* = 116) *n* (%)	E-cigarette user (*n* = 116) *n* (%)	Pearson Chi-Square	*p*-value	
Good					
Normal	93 (80.2)	83 (71.6)	2.354	0.125	
Below normal	23 (19.8)	33 (28.4)			
Competent					
Normal	101 (87.1)	109 (94.0)	3.214	0.073	
Below normal	15 (12.9)	7 (6.0)			
Well-Being					
Normal	105 (90.5)	107 (92.2)	0.219	0.640	
Below normal	11 (9.5)	9 (7.8)			

EQ = Emotional Quotient. Good dimension: Normal score range = 13–23, Competent dimension: Normal score range = 14–20, and Well-being dimension: Normal score range = 9–21. Pearson’s Chi-square test was used to assess differences between groups. *P* < 0.05 indicates statistical significance.

### Dementia risk analysis

An evaluation of dementia risk, based on the proportions presented in Table 4, revealed that 46 e-cigarette users (39.7%) exhibited a higher tendency toward dementia risk compared to 1 non-smoker (0.9%) who showed similar risk levels. The Chi-Square analysis indicated a statistically significant association (*p* < 0.05) between dementia risk and e-cigarette use, suggesting an association between e-cigarette use and increased dementia risk.

**Table 4:** Analysis of the relationship between dementia risk and smoking behavior using the Chi-square test between e-cigarette users and non-smokers.

Potential dementia risk level	Non-smoker (*n* = 116) *n* (%)	E-cigarette user (*n* = 116) *n* (%)	Pearson Chi-Square	*p*-value
Normal	115 (99.1)	70 (60.3)	54.031	< 0.001
At risk for dementia	1 (0.9)	46 (39.7)		

Dementia risk was assessed using the MoCA. The total score is 30 points. A score below 26 indicates cognitive impairment risk, while a score of 26 or higher is considered normal. Pearson’s Chi-square test was used for group comparison. *P* < 0.05 indicates statistical significance.

Eight independent variables related to e-cigarette usage behaviors were included in the model: frequency of e-cigarette use, age of first use, number of times used per day, trend in consumption, frequency of consumption, willingness to quit within one month, willingness to quit within six months, and previous attempts to quit. Additionally, confounding variables were controlled, including demographic factors (gender, age, BMI, education level, occupation, and pre-existing medical conditions) and substance use behaviors (alcohol consumption, drug use, and having family members who smoke). Table 5 were presented the results indicated that, after controlling for confounding variables, two factors were significantly associated with an increased risk of cognitive impairment at the 0.05 significance level. First, participants who did not intend to quit e-cigarettes within the next month had a sixfold higher odds of cognitive impairment compared to those who planned to quit within a month (OR = 6.04, *p* < 0.05). Similarly, participants who did not intend to quit within the next six months had a fourfold higher risk of cognitive impairment compared to those who intended to quit within this period (OR = 4.15, *p* < 0.05). The logistic regression model explained 23.3% of the variance (R² = 0.233) in cognitive impairment risk, suggesting that additional unmeasured factors account for the remaining 76.7% of the variance.

**Table 5:** Binary logistic regression analysis examining the relationship between the likelihood of developing dementia and e-cigarette use behavior (*n* = 116).

**Variables**	**Crude ORs**			**Adjusted ORs**		
	**ORs**	**95% CI**	*P*-value	**ORs**	**95% CI**	**p-value**
E-cigarette consumption rate						
≤ 1 Device/day	1.0			1.0		
> 1 Device/day	1.82	0.649-5.096	0.256	0.27	0.065-1.113	0.070
Age at first use of e-cigarette (Year)						
< 14	0.61	0.227-1.641	0.328	2.03	0.686-6.024	0.201
≥ 14	1.0			1.0		
Frequency of e-cigarette use per day						
< 10 times	1.0			1.0		
≥ 10 times	1.77	0.787-4.020	0.166	3.16	0.996-10.027	0.051
Trend in e-cigarette usage volume						
Decreased	1.0			1.0		
Remained the same	0.87	0.411-1.823	0.704	0.76	0.295-2.449	0.851
Frequency of e-cigarette usage						
Decreased	1.0			1.0		
Remained the same	0.77	0.366-1.627	0.496	0.63	0.278-2.171	0.777
Intention to quit e-cigarette use within the next month						
No	2.83	1.263-6.355	0.012	6.04	1.969-18.536	0.002
Yes	1.0			1.0		
Intention to quit e-cigarette use within the next 6 months						
No	2.29	1.044-5.004	0.039	4.15	1.401-12.311	0.010
Yes	1.0			1.0		
Attempts to quit e-cigarette use						
Never tried	1.12	0.53-2.368	0.767	1.07	0.421-2.730	0.885
Tried	1.0			1.0		

## Discussion

### Dementia risk and e-cigarette use

The findings of this study indicate a statistically significant association between e-cigarette use and increased dementia risk, as demonstrated by the Chi-Square analysis (*p* < 0.05). A higher proportion of e-cigarette users (39.7%) exhibited dementia risk compared to non-smokers (0.9%), highlighting a potential link between nicotine consumption and cognitive decline. These results align with existing literature that suggests nicotine exposure can contribute to neurocognitive deficits by altering synaptic plasticity, increasing oxidative stress, and disrupting neurotransmitter balance in the brain^20,21^. Further analysis using Binary Logistic Regression revealed that participants who did not intend to quit e-cigarettes within one month had a sixfold higher likelihood of cognitive impairment (OR = 6.04, *p* < 0.05), while those who did not intend to quit within six months had a fourfold higher likelihood (OR = 4.15, *p* < 0.05). These findings should be interpreted as associations rather than causal relationships due to the cross-sectional design of the study. This findings suggest that continued e-cigarette use may be associated with cognitive vulnerabilities, potentially related to chronic nicotine exposure and its effects on brain structure and function^22^. The findings are consistent with prior research indicating that persistent nicotine use is associated with memory impairments and an increased risk of neurodegenerative diseases^23^. Despite these significant associations, the logistic regression model accounted for only 23.3% of the variance (R² = 0.233) in dementia risk, implying that additional unmeasured factors contribute to cognitive impairment, such as genetic predisposition, environmental influences, and co-occurring substance use^24^. This highlights the need for cautious interpretation and suggests that dementia risk is likely multifactorial. Future studies should incorporate longitudinal designs to establish temporal relationships and explore biological mechanisms linking e-cigarette use to cognitive decline. As the primary outcome of this study, dementia risk was prioritized in the interpretation of findings, while ADHD symptom tendency and EQ were considered secondary outcomes.

### ADHD symptom tendency and e-cigarette use

The study did not find a statistically significant association between e-cigarette use and ADHD symptom tendency. Several factors may explain this outcome, particularly regarding brain development, cognitive function, and nicotine exposure levels in young adults (18–25 years). Adolescence and early adulthood are critical periods for brain maturation, especially in areas related to impulse control, attention regulation, and executive functioning. The prefrontal cortex, responsible for decision-making and self-regulation, continues developing into the mid-20s^25^. However, by the age range of 18–25, many structural and functional aspects of brain development have stabilized compared to early adolescence. This neural maturity might explain why e-cigarette exposure in this study’s participants did not significantly influence ADHD symptom expression, as their cognitive control systems are more developed than those of younger adolescents who might be more vulnerable to external influences^26^. While nicotine has been associated with cognitive effects such as altered attention and impulse control, the extent of these changes largely depends on the dosage, frequency, and duration of exposure. Studies suggest that chronic high-dose nicotine exposure can affect dopamine pathways, potentially exacerbating ADHD-related symptoms^22^. However, in this study, most participants reported moderate usage patterns, which may not reflect sufficient exposure intensity to produce measurable neurocognitive effects. Additionally, variability in nicotine concentrations across different e-cigarette products makes it difficult to determine a definitive dose-response relationship^27^. Another possible explanation is that individuals in this age group may have developed compensatory mechanisms that mitigate the observable effects of nicotine on attention-related outcomes. Moreover, environmental and lifestyle factors, such as education, cognitive engagement, and social support, could attenuate potential associations^28^. Importantly, the absence of a significant association does not imply the absence of an effect, but rather suggests that any potential relationship may be subtle, context-dependent, or influenced by unmeasured variables. These null findings remain valuable as they contribute to a more balanced understanding of the neurocognitive correlates of e-cigarette use.

### Emotional intelligence (EQ) and e-cigarette use

The present study assessed the relationship between e-cigarette use and EQ levels among young adults. While overall EQ levels were within the normal range across all participants, variations were observed in specific dimensions of EQ, particularly in empathy, decision-making, interpersonal relationships, and emotional well-being. These findings align with previous research suggesting that emotional regulation and social competencies can be influenced by lifestyle behaviors, including substance use^29^.

Regarding the “Good” dimension of EQ, which includes self-regulation, empathy, and responsibility, e-cigarette users and non-users exhibited similar patterns, with empathy and responsibility scoring below normal levels. This aligns with existing literature indicating that nicotine consumption may influence cognitive-affective processing, which can impact social decision-making and responsibility^30^. However, the statistical analysis revealed no significant association between e-cigarette use and this dimension (*p* > 0.05). In the “Competent” dimension, which assesses motivation, decision-making, and relationship-building skills, a greater proportion of non-smokers exhibited below-normal EQ scores compared to e-cigarette users. Although prior studies have linked nicotine use with altered reward sensitivity and motivational control^31^, this difference was not statistically significant. This pattern may reflect underlying social or contextual factors rather than true differences in emotional intelligence, particularly given that e-cigarette use often occurs in social environments influenced by peer interaction. Similarly, in the “Well-Being” dimension, a higher proportion of non-smokers exhibited below-normal EQ scores. While e-cigarette users maintained normal overall EQ levels in this dimension, some subcomponents such as self-satisfaction and inner peace were lower than expected. Some studies suggest that nicotine consumption may temporarily alleviate stress and anxiety, leading to self-reported emotional stability despite potential long-term consequences^32^. Overall, the lack of statistically significant associations across EQ dimensions suggests that e-cigarette use may not be a primary determinant of emotional intelligence in this population, although indirect psychosocial factors may still play a role. The absence of significant associations for ADHD symptom tendency and EQ should be interpreted in the context of these being secondary outcomes, and may suggest that e-cigarette use is not uniformly associated with all domains of neuropsychological functioning.

### Participant characteristics of e-cigarette users

This study found that e-cigarette use was more prevalent among males than females, consistent with previous research indicating that males are more likely to engage in smoking behaviors due to social norms and gender-related influences^33^. In Thai society, traditional values often encourage females to adopt more conservative behaviors, while males face fewer restrictions regarding smoking. This aligns with findings from other studies suggesting that social acceptance and peer influence contribute to higher smoking rates among males compared to females^34^. Most e-cigarette users in this study were between 20 and 25 years old, with many initiating use between the ages of 17 to 19. These findings align with national statistics indicating that adolescents and young adults constitute the primary demographic for e-cigarette use in Thailand. Research suggests that this age group is particularly vulnerable due to increased exposure to peer pressure, curiosity, and the perceived social acceptability of vaping^35^. Furthermore, most e-cigarette users were students, which is consistent with international research showing that university students have a high likelihood of engaging in e-cigarette use^36^. Environmental and familial influences also played a significant role in e-cigarette use, as many participants reported having family members who smoked or vaped. This supports prior research suggesting that adolescents with family members who smoke are more likely to engage in vaping due to modeled behavior and easier access to e-cigarette products^37^.

## Limitations

This study did not assess nicotine dependence using validated instruments such as the Penn State Nicotine Dependence Index (PSNDI), which may act as a confounding factor. Therefore, it is not possible to determine whether the observed associations are attributable to e-cigarette use itself or differences in nicotine dependence levels. Future studies should incorporate standardized measures of nicotine dependence.

In addition, this study was conducted in a single urban district (Lat Krabang, Bangkok), which may limit the generalizability of the findings to other populations in Thailand. However, the study provides important preliminary evidence from an underrepresented context and contributes to the limited body of research on e-cigarette use and neurocognitive outcomes in Southeast Asia. These findings may help inform future large-scale and nationally representative studies. Although the study was conducted in a single district, the findings may reflect patterns observed in other urban areas of Thailand, where e-cigarette use among young adults is increasing. Therefore, these results may provide preliminary insights to inform public health strategies and early prevention efforts at the national level.

Furthermore, the classification of gender/sexual identity was based on self-reported categories (male, female, or LGBTQ+) and did not distinguish between sex assigned at birth, gender identity, and sexual orientation. Therefore, this variable should be interpreted with caution, as it may not fully capture the complexity of gender-related differences. Future studies should incorporate more detailed and standardized measures to better differentiate these constructs.

## Conclusions

This study provides valuable insights into the associations between e-cigarette use and ADHD symptoms, emotional intelligence (EQ) levels, and dementia risk among young adults. While no significant relationship was found between e-cigarette use and ADHD symptom prevalence, potential explanations include the participants’ age range (18–25 years), brain maturation stages, and variability in nicotine exposure levels. Regarding EQ, although specific dimensions such as empathy, decision-making, and well-being showed variations, no direct causal relationship between e-cigarette use and overall EQ levels was established. However, the results suggest that lifestyle factors, peer influence, and psychosocial contexts may contribute to emotional regulation differences in e-cigarette users and non-users.

Notably, a significant association was observed between e-cigarette use and dementia risk, with logistic regression analysis indicating that continued e-cigarette use was associated with a higher likelihood of cognitive impairment. Participants who did not intend to quit within one month or six months exhibited significantly higher odds of cognitive decline, highlighting the potential long-term neurological risks of persistent nicotine exposure. Despite this significant finding, the model accounted for only a portion of the variance, suggesting the influence of additional genetic, environmental, and behavioral factors.

Given these findings, public health interventions should prioritize awareness campaigns on the cognitive risks of e-cigarette use, promote smoking cessation programs, and implement stricter regulations on e-cigarette accessibility. Future research should focus on longitudinal studies to establish causal relationships, incorporate diverse demographic and lifestyle factors, and investigate potential neurobiological mechanisms underlying the observed cognitive effects. Understanding the long-term effects of e-cigarette use remains critical in developing targeted policies and interventions to mitigate associated health risks. The findings should be interpreted with caution as the study was conducted in a single urban district and may not be generalizable to all Thai young adults.

## Supplementary Information

Below is the link to the electronic supplementary material.

Supplementary Material 1

Supplementary Material 2

## Data Availability

This published article contains all of the data created or examined during this investigation.

## References

[1] Lumen Lerning. Brain Development During Adolescence. https://courses.lumenlearning.com/wm-lifespandevelopment/chapter/brain-development-during-adolescence/

[2] Kanyaphat Setchoduk. Electronic Cigarette: a Youths’ Health Threat. *J. Health Sci. Thail. (JHS)*. **25** (6), 1075–1083 (2016).

[3] Harrell, M. B., Mantey, D. S., Chen, B., Kelder, S. H. & Barrington-Trimis, J. Impact of the e-cigarette era on cigarette smoking among youth in the United States: A population-level study. *Prev. Med.* **164**, 107265 (2022). 36152819 10.1016/j.ypmed.2022.107265 PMC10381788

[4] Chardsumon Prutipinyo, V. & Pipattanachat, S. H. *Hidden Dangers of E-cigarette*. (2019). https://www.trc.or.th/th/attachments/article/395/%E0%B8%A0%E0%B8%B1%E0%B8%A2%E0%B8%A3%E0%B9%89%E0%B8%B2%E0%B8%A2%20%E0%B8%8B%E0%B9%88%E0%B8%AD%E0%B8%99%E0%B9%80%E0%B8%A3%E0%B9%88%E0%B8%99%20%E0%B8%9A%E0%B8%B8%E0%B8%AB%E0%B8%A3%E0%B8%B5%E0%B9%88%E0%B9%84%E0%B8%9F%E0%B8%9F%E0%B9%89%E0%B8%B2.pdf

[5] Dalrymple, R. A., Maxwell, L. M., Russell, S. & Duthie, J. NICE guideline review: Attention deficit hyperactivity disorder: Diagnosis and management (NG87). *Arch. Dis. Child. Educ. Pract. Ed.* **105**(5), 289–293 (2020). 31776172 10.1136/archdischild-2019-316928

[6] Peterson, L. A. & Hecht, S. S. Tobacco, e-cigarettes, and child health. *Curr. Opin. Pediatr.* **29**(2), 225–230 (2017). 28059903 10.1097/MOP.0000000000000456 PMC5598780

[7] Le Foll, B. et al. Tobacco and nicotine use. *Nat. Rev. Dis. Primers* **8**(1), 19 (2022). 35332148 10.1038/s41572-022-00346-w

[8] Wawryk-Gawda, E., Lis-Sochocka, M., Chylińska-Wrzos, P., Budzyńska, B. & Jodłowska-Jędrych, B. The impact of traditional cigarettes and e-cigarettes on the brain. *Neuroscience of Nicotine*. Elsevier; :25–32. (2019).

[9] Department of Mental Health MoPH & Thailand (18–60) Department of Mental Health, Ministry of Public Health, Thailand. https://dmh-elibrary.org/items/show/42

[10] Goleman, D. *Working with emotional intelligence* (Bantam, 1998).

[11] World Health Organization; WHO & Dementia (2021). https://www.who.int/news-room/fact-sheets/detail/dementia

[12] Lat Krabang District Office. General Information. https://webportal.bangkok.go.th/ladkrabang/page/sub/3678/%E0%B8%82%E0%B9%89%E0%B8%AD%E0%B8%A1%E0%B8%B9%E0%B8%A5%E0%B8%97%E0%B8%B1%E0%B9%88%E0%B8%A7%E0%B9%84%E0%B8%9B.

[13] Bangkok Metropolitan Administration. Statistical profile of Bangkok Metropolitan Administration,. (2016). https://apps.bangkok.go.th/info_gidsedbkk/m.info/bkkstat/stat_2559_eng.pdf

[14] Thailand Board of Investment. industrial Estates in Thailand. https://www.boi.go.th/index.php?language=en&page=industral_estate_in_thailand&utm_source=chatgpt.com

[15] Reff, J. & Baschnagel, J. S. The role of affective urgency and emotion regulation in vaping susceptibility. *Addict. Behav. Rep.* **14**, 100355 (2021). 34136632 10.1016/j.abrep.2021.100355 PMC8181786

[16] Kessler, R. C. et al. The World Health Organization Adult ADHD Self-Report Scale (ASRS): a short screening scale for use in the general population. *Psychol. Med.* **35** (2), 245–256 (2005). 15841682 10.1017/s0033291704002892

[17] Kahn, K. B., Barczak, G. & Moss, R. Perspective: Establishing an NPD best practices framework. *J. Prod. Innov. Manag.* **23**(2), 106–116 (2006).

[18] Nasreddine, Z. S. et al. The Montreal Cognitive Assessment, MoCA: a brief screening tool for mild cognitive impairment. *J. Am. Geriatr. Soc.* **53** (4), 695–699 (2005). 15817019 10.1111/j.1532-5415.2005.53221.x

[19] Tangwongchai, S. et al. In thai Nationals, the ApoE4 allele affects multiple domains of neuropsychological, biobehavioral, and social functioning thereby contributing to Alzheimer’s disorder, while the ApoE3 allele protects against neuropsychiatric symptoms and psychosocial deficits. *Mol. Neurobiol.* **55**, 6449–6462 (2018). 29307083 10.1007/s12035-017-0848-0

[20] Ali, N., Xavier, J., Engur, M., Mohanan, P. & de la Serna, J. B. The impact of e-cigarette exposure on different organ systems: A review of recent evidence and future perspectives. *J. Hazard. Mater.* **457**, 131828 (2023). 37320902 10.1016/j.jhazmat.2023.131828

[21] Swan, G. E. & Lessov-Schlaggar, C. N. The effects of tobacco smoke and nicotine on cognition and the brain. *Neuropsychol. Rev.* **17**, 259–273 (2007). 17690985 10.1007/s11065-007-9035-9

[22] Besson, M. & Forget, B. Cognitive dysfunction, affective states, and vulnerability to nicotine addiction: A multifactorial perspective. *Front. Psychiatry* **7**, 160 (2016). 27708591 10.3389/fpsyt.2016.00160 PMC5030478

[23] Durazzo, T. C., Meyerhoff, D. J. & Nixon, S. J. Chronic cigarette smoking: Implications for neurocognition and brain neurobiology. *Int. J. Environ. Res. Public Health* **7**(10), 3760–3791 (2010). 21139859 10.3390/ijerph7103760 PMC2996190

[24] Peters, R. et al. Smoking, dementia and cognitive decline in the elderly, a systematic review. *BMC Geriatr.* **8**, 1–7 (2008). 19105840 10.1186/1471-2318-8-36 PMC2642819

[25] Casey, B. J., Getz, S. & Galvan, A. The adolescent brain. *Dev. Rev.* **28**(1), 62–77 (2008). 18688292 10.1016/j.dr.2007.08.003 PMC2500212

[26] Giedd, J. N. The amazing teen brain. *Sci. Am.* **312**(6), 32–37 (2015). 26336683 10.1038/scientificamerican0615-32

[27] Hammond, D., Reid, J. L., Cole, A. G. & Leatherdale, S. T. Electronic cigarette use and smoking initiation among youth: A longitudinal cohort study. *CMAJ* **189**(43), E1328–E1336 (2017). 29084759 10.1503/cmaj.161002 PMC5662449

[28] Volkow, N. D., Koob, G. F. & McLellan, A. T. Neurobiologic advances from the brain disease model of addiction. *Evaluating Brain Disease Model. Addiction* :25–34. (2022). 10.1056/NEJMra1511480 PMC6135257 26816013

[29] Piko, B. F. & Kovács, E. Do parents and school matter? Protective factors for adolescent substance use. *Addict. Behav.* **35**(1), 53–56 (2010). 19720468 10.1016/j.addbeh.2009.08.004

[30] Schoenbaum, G., Roesch, M. R., Stalnaker, T. A. & Takahashi, Y. K. A new perspective on the role of the orbitofrontal cortex in adaptive behaviour. *Nat. Rev. Neurosci.* **10**(12), 885–892 (2009). 19904278 10.1038/nrn2753 PMC2835299

[31] Barr, R. S., Pizzagalli, D. A., Culhane, M. A., Goff, D. C. & Evins, A. E. A single dose of nicotine enhances reward responsiveness in nonsmokers: Implications for development of dependence. *Biol. Psychiatry* **63**(11), 1061–1065 (2008). 17976537 10.1016/j.biopsych.2007.09.015 PMC2441863

[32] Benowitz, N. L. Nicotine addiction. *N. Engl. J. Med.* **362**(24), 2295–2303 (2010). 20554984 10.1056/NEJMra0809890 PMC2928221

[33] Xiao, D. et al. Prevalence and risk factors of small airway dysfunction, and association with smoking, in China: findings from a national cross-sectional study. *Lancet Respiratory Med.* **8** (11), 1081–1093 (2020). 10.1016/S2213-2600(20)30155-7 32598906

[34] Syamlal, G., Mazurek, J. M. & Dube, S. R. Gender differences in smoking among US working adults. *Am. J. Prev. Med.* **47** (4), 467–475 (2014). 25049215 10.1016/j.amepre.2014.06.013 PMC4542001

[35] Thai Health. Thailand’s Commitment in COP (Conference of Parties) & Responses to Climate Change. 1 ed. Institute for Population and Social Research, Mahidol University; no. 599. (2023). https://www.thaihealth.or.th/wp-content/uploads/2023/11/Thai-Health-2023.pdf

[36] Patel, D. et al. Reasons for current E-cigarette use among US adults. *Prev. Med.* **93**, 14–20 (2016). 27612572 10.1016/j.ypmed.2016.09.011 PMC5316292

[37] Roh, E. J., Chen-Sankey, J. C. & Wang, M. Q. Electronic nicotine delivery system (ENDS) use patterns and its associations with cigarette smoking and nicotine addiction among Asian Americans: Findings from the national adult tobacco survey (NATS) 2013–2014. *J. Ethn. Subst. Abuse* **21**(1), 253–271 (2022). 32459579 10.1080/15332640.2020.1747039 PMC7704701

