# Supplementary material for: The impact of e-cigarette use on cognitive function, emotional intelligence, and dementia risk in adolescents and young adults
Source: Sci Rep. 2026 Apr 12;16:17015. doi: 10.1038/s41598-026-48579-z (PMC13230882; doi:10.1038/s41598-026-48579-z)
Supplement: Supplementary file 1 — Supplementary Material 1 [file 41598_2026_48579_MOESM1_ESM.docx]

**Supplementary Material**


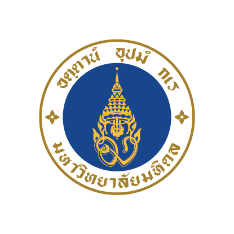


**Questionnaire**

**Research title “The Impact of E-Cigarette Use on Cognitive Function, Emotional Intelligence, and Dementia Risk in Adolescents and Young Adults”**

CODE Date/Month/Year Data reporter

**Instructions**

This questionnaire is part of the courses “Research Methodology in Public Health” and “Term Paper” under the Bachelor of Public Health Program (Community Public Health), Nakhon Sawan Campus Project, Mahidol University. The objective of this study is to assess the tendency of attention-deficit/hyperactivity disorder (ADHD) symptoms, levels of emotional intelligence (EQ), and the risk of cognitive impairment (dementia) among e-cigarette users compared with non-smokers.

All personal information obtained from this questionnaire will be kept strictly confidential. The data will be analyzed and presented in aggregate form only, without any identification of individual respondents. You are kindly requested to complete this questionnaire honestly and thoroughly, as your responses are highly valuable for the accuracy and quality of this research.


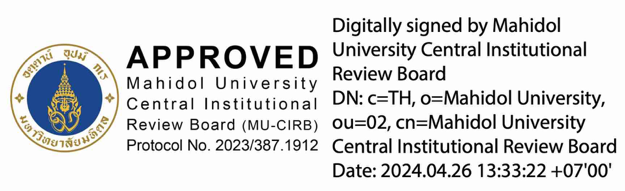
The questionnaire consists of six sections, including both open-ended and closed-ended questions.

**Sections 1–4: Self-administered components**

These sections were completed independently by the participants as follows:

**Section 1:** General Information

This section collects basic demographic and personal information of the respondents.

**Section 2:** Tobacco, Alcohol, and Substance Use

This section assesses the respondents’ history and patterns of tobacco use, alcohol consumption, and substance use.

**Section 3:** E-Cigarette Use Behavior

This section examines the respondents’ behavioral patterns, history, and related information regarding e-cigarette use.

**Section 4:** Emotional Intelligence (EQ) Assessment

This section consists of an Emotional Intelligence (EQ) assessment developed by the Department of Mental Health, Thailand. The items are designed to evaluate emotional awareness, emotional regulation, and expression through various statements reflecting feelings and emotional responses.

**Sections 5–6: Interviewer-administered components**

These sections were administered and recorded by trained researchers to ensure accuracy and consistency as follows:

**Section 5:** ADHD Symptom Screening (ASRS-v1.1; 18 items)

This section assesses symptoms of Attention-Deficit/Hyperactivity Disorder (ADHD) using the Adult ADHD Self-Report Scale (ASRS-v1.1). It serves as a screening tool for identifying ADHD symptom tendency based on criteria from the Diagnostic and Statistical Manual of Mental Disorders, Fifth Edition (DSM-5), developed by the American Psychiatric Association. The instrument is also consistent with screening approaches recommended in reports by the World Health Organization (WHO).

**Section 6:** Cognitive Assessment for Dementia (MoCA: Montreal Cognitive Assessment)

This section evaluates cognitive function in adults using the Montreal Cognitive Assessment (MoCA). It is a standardized screening tool specifically designed to detect early signs of cognitive impairment and dementia.

**Instructions:** Please complete all items by filling in the blanks and marking (✓) in the appropriate boxes (☐). Select the responses that best reflect your personal characteristics and actual situation.

**Section 1: General Information**

1. Sex: ☐ 1. Male ☐ 2. Female ☐ 3. LGBTQ+
2. Age: ______ years
3. Weight: ______ kg
4. Height: ______ cm
5. Highest education level:
 ☐ No formal education

☐ Primary

☐ Secondary/Vocational

☐ Diploma

☐ Bachelor’s degree or higher
6. Marital status:

☐ 1. Single (skip to Question no.8) ☐ 2. Married ☐ 3. Divorced

☐ 4. Widowed ☐ 5. Separated
7. Number of children: ☐ 1. None ☐ 2. Yes, ______ children
8. Household size: ☐ 1. 1–3 person ☐ 2. 4–6 person ☐ 3. More than 6 person
9. Residence:

☐ 1. House ☐ 2. On-campus dormitory ☐ 3. Off-campus dormitory

☐ 4. Other: please specify ______
10. Chronic disease:

☐ None

☐ Yes: please specify ______
11. Occupation:

Primary Occupation:

☐ 1. Student ☐ 2. Business ☐ 3. Government

☐ 4. Private employee ☐ 5. Farmer ☐ 6. Unemployed

☐ 7. Other, please specify ______

Secondary Occupation (s); If any, please specify:

1.

2.
12. Monthly income:

☐ 1. <5,000 THB/Month

☐ 2. 5,001–10,000 THB/Month

☐ 3. >10,000 THB/Month

☐ 4. Other, please specify

**Section 2: Tobacco, Alcohol, and Substance Use**

1. Have you ever smoked cigarettes or used e-cigarettes?

☐ 1. Never (Skip to Question no.3)
☐ 2. Tried (_____ times)
☐ 3. Currently smoke conventional cigarettes (_____ cigarettes/day)
☐ 4. Currently use e-cigarettes (_____ pods or bottles/day)
☐ 5. Former smoker (quit for _____ years)

1.1 Smoking/Vaping Behavior

☐ 1. Conventional cigarettes only
☐ 2. E-cigarettes only
☐ 3. Both conventional cigarettes and e-cigarettes (dual use)

2. At what age did you first smoke?

☐ 1. <14 years ☐ 2. 14–16 years ☐ 3. 17–19 years
☐ 4. 20–22 years ☐ 5. 23–25 years

3. Have you ever consumed alcoholic beverages in your lifetime?

☐ 1. Never ☐ 2. Currently drink (_____ bottles/day)
☐ 3. Tried once ☐ 4. Former drinker (quit for _____ years)

4. Have you ever used illicit drugs?

☐ 1. Never used ☐ 2. Currently use (_____ times/day)
☐ 3. Former user (quit for _____ years) ☐ 4. Prefer not to disclose

5. Do any of your family members smoke?

☐ 1. No ☐ 2. Yes (_____ persons; please specify: ____________________)

6. Reasons for smoking or using e-cigarettes

(Select all that apply; if never used, skip to Section 4)

☐ 1. Curiosity / experimentation
☐ 2. Peer influence (friends or surrounding individuals encouraged use)
☐ 3. Stress relief
☐ 4. Socialization purposes
☐ 5. Attempted to quit but unsuccessful
☐ 6. Perception that e-cigarettes are less harmful than conventional cigarettes
☐ 7. Use e-cigarettes to quit conventional smoking
☐ 8. Desire to avoid cigarette odor
☐ 9. Flavor or aroma
☐ 10. Product design and appearance
☐ 11. Interest in device features and functionality
☐ 12. Convenience of use
☐ 13. Perceived lower cost compared to conventional cigarettes

7. Time periods of smoking or e-cigarette use (Select all that apply)

☐ 1. 04:01–08:00
☐ 2. 08:01–12:00
☐ 3. 12:01–16:00
☐ 4. 16:01–20:00
☐ 5. 20:01–00:00
☐ 6. 00:01–04:00

8. Monthly expenditure on cigarettes or e-cigarettes

☐ 1. <100 THB/month ☐ 2. 100–500 THB/month

☐ 3. 500–1,000 THB/month ☐ 4. 1,000–5,000 THB/month

☐ 5. 5,000–10,000 THB/month ☐ 6. >10,000 THB/month

**Section 3:** **E-Cigarette Use Behavior**

(If you have never used e-cigarettes, please skip to Section 4)

1. Frequency of e-cigarette use per day

☐ 1. Once per day ☐ 2. 2–5 times per day

☐ 3. 6–10 times per day ☐ 4. More than 10 times per day

2. Brand of e-cigarette liquid (e-liquid) used. Please specify: _____________________________

3. Situations or times when e-cigarettes are used (Select all that apply)

☐ 1. While consuming alcohol
☐ 2. Immediately after waking up
☐ 3. When socializing or spending time with friends
☐ 4. Before work/class
☐ 5. During work/class
☐ 6. After work/class
☐ 7. Before meals
☐ 8. During meals
☐ 9. After meals
☐ 10. During social gatherings or parties
☐ 11. No specific pattern

4. Locations where e-cigarettes are used

☐ 1. At home or dormitory

☐ 2. At a friend’s home or dormitory
☐ 3. Entertainment venues (e.g., bars, clubs)

☐ 4. Educational institutions
☐ 5. Public places
☐ 6. No specific location

5. People you usually use e-cigarettes with (Select all that apply)

☐ 1. Close friends

☐ 2. Alone
☐ 3. Seniors or juniors (e.g., upperclassmen/lowerclassmen)

☐ 4. Family members
☐ 5. Partner
☐ 6. Others (please specify): __________

6. Sources of e-cigarettes (Select all that apply)

☐ 1. Purchased online from websites
☐ 2. LINE application
☐ 3. Facebook
☐ 4. Asked someone else to purchase
☐ 5. Purchased second-hand from friends or acquaintances
☐ 6. Borrowed from friends
☐ 7. Purchased from general retail stores
☐ 8. Others (please specify): __________

7. Channels through which you have been exposed to e-cigarette advertising or promotion

☐ 1. Social media platforms (e.g., Facebook, LINE, Shopee, Lazada)
☐ 2. Peer groups or friends
☐ 3. Dedicated e-cigarette sales websites
☐ 4. Direct purchase from sellers (no storefront or official distributor)
☐ 5. Imported from abroad (e.g., pre-order or via acquaintances)
☐ 6. Physical e-cigarette shops / street vendors / markets
☐ 7. Others (please specify): __________

8. Change in the amount of e-cigarette use since initiation

☐ 1. Decreased ☐ 2. Remained the same ☐ 3. Increased

9. Change in the frequency of e-cigarette use since initiation

☐ 1. Decreased ☐ 2. Remained the same ☐ 3. Increased

10. Intention to quit e-cigarette use within the next 1 month

☐ 1. No ☐ 2. Yes ☐ 3. Uncertain

11. Intention to quit e-cigarette use within the next 6 months

☐ 1. No ☐ 2. Yes ☐ 3. Uncertain

12. Have you ever attempted to quit using e-cigarettes?

☐ 1. Never ☐ 2. Yes, ______ times ☐ 3. Quit but relapsed

**Section 4:** **Emotional Intelligence (EQ) Assessment**

This questionnaire consists of statements related to emotions and feelings expressed in various situations. Some statements may not exactly match your personal experience; however, please select the response that best reflects yourself. There are no right or wrong answers, and no responses are considered good or bad. Please answer honestly and respond to all items. Your responses will help you better understand yourself and support future self-development.

| **No.** | **Question** | **Not true** | **Sometimes true** | **Quite true** | **Very true** |
| --- | --- | --- | --- | --- | --- |
| 1 | When I feel angry or upset, I am aware of what is happening to me. |  |  |  |  |
| 2 | I cannot identify what makes me feel angry. |  |  |  |  |
| 3 | When I feel frustrated, I often lose control of my emotions. |  |  |  |  |
| 4 | I am able to wait patiently to achieve my desired goals. |  |  |  |  |
| 5 | I tend to react strongly to minor problems. |  |  |  |  |
| 6 | When forced to do something I dislike, I can explain my reasons until others accept them. |  |  |  |  |
| 7 | I can notice when people close to me experience emotional changes. |  |  |  |  |
| 8 | I do not care about the suffering of people I do not know. |  |  |  |  |
| 9 | I cannot accept when others behave differently from what I expect. |  |  |  |  |
| 10 | I can accept that others may have reasons to be dissatisfied with my actions. |  |  |  |  |
| 11 | I feel that others demand too much attention. |  |  |  |  |
| 12 | Even when I am busy, I am willing to listen to others’ problems. |  |  |  |  |
| 13 | It is normal to take advantage of others when the opportunity arises. |  |  |  |  |
| 14 | I appreciate the kindness that others show toward me. |  |  |  |  |
| 15 | When I make a mistake, I am able to apologize. |  |  |  |  |
| 16 | I find it difficult to accept others’ mistakes. |  |  |  |  |
| 17 | Even if it means sacrificing personal benefits, I am willing to act for the greater good. |  |  |  |  |
| 18 | I feel uncomfortable doing things for others. |  |  |  |  |
| 19 | I do not know what I am good at. |  |  |  |  |
| 20 | Even when tasks are difficult, I am confident that I can complete them. |  |  |  |  |
| 21 | When I fail, I feel discouraged. |  |  |  |  |
| 22 | I feel valuable when I perform tasks to the best of my ability. |  |  |  |  |
| 23 | When facing obstacles or disappointment, I do not give up. |  |  |  |  |
| 24 | When I start something, I often fail to complete it. |  |  |  |  |
| 25 | I try to identify the true cause of problems rather than making assumptions. |  |  |  |  |
| 26 | I often do not know what makes me unhappy. |  |  |  |  |
| 27 | I find decision-making difficult. |  |  |  |  |
| 28 | When doing multiple tasks at once, I can decide what to do first. |  |  |  |  |
| 29 | I feel uncomfortable when interacting with strangers. |  |  |  |  |
| 30 | I cannot tolerate social rules that conflict with my habits. |  |  |  |  |
| 31 | I can easily get to know new people. |  |  |  |  |
| 32 | I have many close friends whom I have known for a long time. |  |  |  |  |
| 33 | I am not confident in expressing my needs to others. |  |  |  |  |
| 34 | I can achieve what I want without causing problems for others. |  |  |  |  |
| 35 | I find it difficult to argue with others, even when I have good reasons. |  |  |  |  |
| 36 | When I disagree with others, I can explain my reasons in a way they accept. |  |  |  |  |
| 37 | I feel inferior to others. |  |  |  |  |
| 38 | I perform well regardless of the role I take. |  |  |  |  |
| 39 | I can complete assigned tasks effectively. |  |  |  |  |
| 40 | I lack confidence when performing difficult tasks. |  |  |  |  |
| 41 | Even in difficult situations, I remain hopeful. |  |  |  |  |
| 42 | There is always a solution to every problem. |  |  |  |  |
| 43 | When I feel stressed, I can turn it into a more relaxing or enjoyable situation. |  |  |  |  |
| 44 | I enjoy activities during weekends and holidays. |  |  |  |  |
| 45 | I feel dissatisfied when others receive better things than I do. |  |  |  |  |
| 46 | I am satisfied with who I am. |  |  |  |  |
| 47 | I do not know what to do when I feel bored. |  |  |  |  |
| 48 | When I have free time, I engage in activities that I enjoy. |  |  |  |  |
| 49 | When I feel distressed, I have ways to manage and relax my emotions. |  |  |  |  |
| 50 | I am able to relax even when I am physically tired. |  |  |  |  |
| 51 | I cannot feel happy unless I get everything I want. |  |  |  |  |
| 52 | I often feel distressed about minor issues. |  |  |  |  |

**Section 5:** **ADHD Symptom Screening (ASRS-v1.1; 18 items)**

Please place a check mark (✓) in the box that best reflects your feelings and behaviors over the past 6 months. After completing this questionnaire, please return it to your attending physician for evaluation.

| **Question** | **Never** | **Rarely** | **Sometimes** | **Often** | **Very Often** |
| --- | --- | --- | --- | --- | --- |
| 1. After completing the most difficult part of a task, how often do you have difficulty finishing the remaining parts? |  |  |  |  |  |
| 2. How often do you have difficulty organizing tasks due to poor planning or management? |  |  |  |  |  |
| 3. How often do you forget appointments or obligations? |  |  |  |  |  |
| 4. How often do you avoid or delay starting tasks that require a lot of thought? |  |  |  |  |  |
| 5. How often do you fidget or move your hands or feet when you have to sit for a long time? |  |  |  |  |  |
| 6. How often do you feel overly active and compelled to do things, as if driven by a motor? |  |  |  |  |  |
| 7. How often do you make careless mistakes when working on boring or difficult tasks? |  |  |  |  |  |
| 8. How often do you have difficulty maintaining attention when doing repetitive or uninteresting work? |  |  |  |  |  |
| 9. How often do you have difficulty concentrating on what others are saying to you, even when they are speaking directly to you? |  |  |  |  |  |
| 10. How often do you misplace things or have difficulty finding items at home or at work? |  |  |  |  |  |
| 11. How often are you distracted by activities or noises around you? |  |  |  |  |  |
| 12. How often do you leave your seat in situations where you are expected to remain seated (e.g., meetings)? |  |  |  |  |  |
| 13. How often do you feel restless or fidgety? |  |  |  |  |  |
| 14. How often do you have difficulty relaxing or unwinding during your free time? |  |  |  |  |  |
| 15. How often do you find yourself talking excessively in social situations? |  |  |  |  |  |
| 16. When in a conversation, how often do you interrupt others before they finish speaking? |  |  |  |  |  |
| 17. How often do you have difficulty waiting your turn in situations that require waiting? |  |  |  |  |  |
| 18. How often do you interrupt others when they are busy? |  |  |  |  |  |


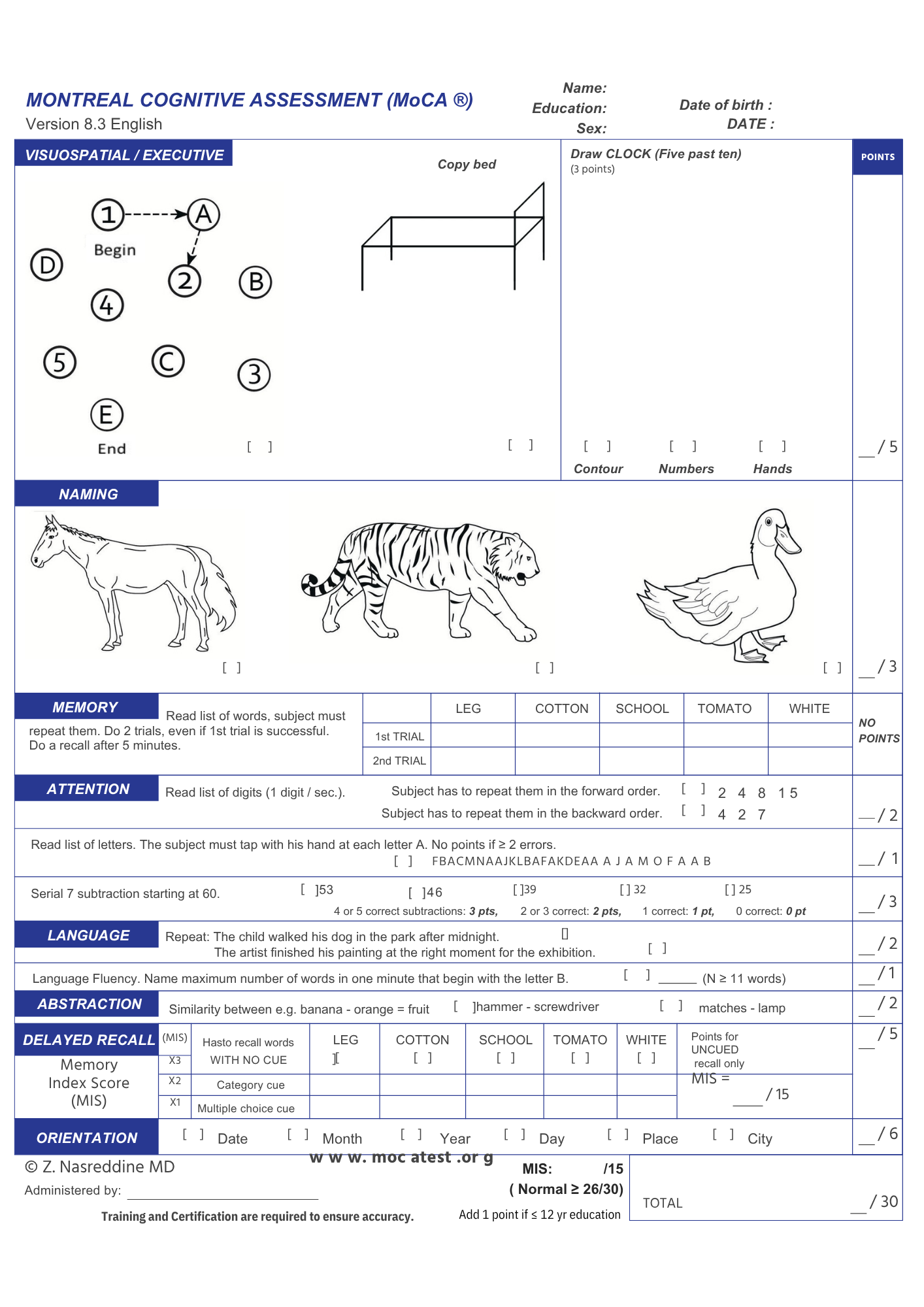
**Section 6:** **Cognitive Assessment for Dementia (MoCA: Montreal Cognitive Assessment)**
