# Supplementary material for: The impact of e-cigarette use on cognitive function, emotional intelligence, and dementia risk in adolescents and young adults
Source: Sci Rep. 2026 Apr 12;16:17015. doi: 10.1038/s41598-026-48579-z (PMC13230882; doi:10.1038/s41598-026-48579-z)
Supplement: Supplementary file 2 — Supplementary Material 2 [file 41598_2026_48579_MOESM2_ESM.docx]

**Supplementary Table**

**Supplementary Table S1** Frequency and percentage of smoking, alcohol, and substance use among participants (n = 232)

| **Variables** | | **Non-smoker (%)**  **(n= 116)** | **E-cigarette user (%)**  **(n= 116)** | **All participants (%)**  **(n = 232)** |
| --- | --- | --- | --- | --- |
| Alcohol drinking behavior | | | | |
|  | Never drank | 45 (38.8) | 10 (8.6) | 55 (23.7) |
|  | Currently drinking | 65 (56.0) | 104 (89.7) | 169 (72.8) |
|  | Former drinker | 6 (5.2) | 2 (1.7) | 8 (3.4) |
| Substance use behavior | | | | |
|  | Never used | 116 (100) | 105 (90.5) | 221 (95.3) |
|  | Currently using | 0 (0) | 2 (1.7) | 2 (0.9) |
|  | Previously used but quit | 0 (0) | 9 (7.8) | 9 (3.9) |
| Presence of a smoking family member | | | | |
|  | No | 60 (51.7) | 54 (46.6) | 114 (49.1) |
|  | Yes | 56 (48.3) | 62 (53.4) | 118 (50.9) |

Note: Values are presented as frequency (%).

**Supplementary Table S2** Characteristics of smoking behavior among the e-cigarette user group (n = 116)

| **E-cigarette use behavior** | | **n (%)** |
| --- | --- | --- |
| E-cigarette consumption rate | |  |
|  | ≤ 1 Device/day | 95 (81.9) |
|  | > 1 Device/day | 21 (18.1) |
| Age at first use of e-cigarette (Year) | |  |
|  | < 14 | 22 (19.0) |
|  | 14-16 | 27 (23.3) |
|  | 17-19 | 39 (33.6) |
|  | 20-22 | 24 (20.7) |
|  | 23-25 | 4 (3.4) |
| Initial reasons for e-cigarette use | |  |
|  | Curiosity and desire to try e-cigarettes | 69 (16.8) |
|  | Peer influence or recommendations from others | 37 (9.0) |
|  | Stress or emotional coping | 38 (9.2) |
|  | Socialization or fitting in | 22 (5.4) |
|  | Attempted cessation of smoking but unsuccessful | 14 (3.4) |
|  | Belief that e-cigarettes are less harmful than traditional cigarettes | 44 (10.7) |
|  | Intention to quit smoking traditional cigarettes using e-cigarettes | 21 (5.1) |
|  | Desire to smoke without the odor of traditional cigarettes | 32 (7.8) |
|  | Preference for taste or scent | 55 (13.4) |
|  | Attraction to the appearance and design of e-cigarettes | 12 (2.9) |
|  | Interest in the functionality and features of e-cigarettes | 12 (2.9) |
|  | Convenience of use | 41 (10.0) |
|  | Perception that e-cigarettes are less expensive than traditional cigarettes | 14 (3.4) |
| Time of day for smoking or e-cigarette use | |  |
|  | 04.01-08.00 AM | 19 (6.4) |
|  | 08.01-12.00 AM | 56 (18.9) |
|  | 01.01-04.00 PM | 55 (18.5) |
|  | 04.01-08.00 PM | 62 (20.9) |
|  | 08.01-12.00 PM | 75 (25.3) |
|  | 00.01-04.00 AM | 30 (10.1) |
| Expenditure on smoking or e-cigarette use per month (THB/Month) | |  |
|  | < 100 (~2.97 USD) | 31 (26.7) |
|  | 100-500 (~2.97–14.83 USD) | 49 (42.2) |
|  | 501-1,000 (~14.93–29.66 USD) | 25 (21.6) |
|  | 1,001-5,000 (~29.99–148.28 USD) | 9 (7.8) |
|  | 5,001-10,000 (~148.56–296.55 USD) | 0 (0) |
|  | > 10,000 (~296.55 USD) | 2 (1.7) |

Note: Values are presented as frequency (%) based on multiple response items.

**Supplementary Table S3** Frequency and Percentage of Smoking Behavior Characteristics Among the Sample Group (n = 116)

| **E-cigarette usage behavior** | | **n (%)** |
| --- | --- | --- |
| Frequency of e-cigarette use per day | |  |
|  | Once | 16 (13.8) |
|  | 2-5 times | 42 (36.2) |
|  | 6-10 times | 19 (16.4) |
|  | More than 10 times | 39 (33.6) |
| Time of e-cigarette use | |  |
|  | While drinking alcohol | 76 (26.8) |
|  | Immediately after waking up | 7 (2.5) |
|  | While socializing with friends | 39 (13.7) |
|  | Before work/school | 19 (6.7) |
|  | During work/school | 8 (2.8) |
|  | After work/school | 21 (7.4) |
|  | Before meals | 5 (1.8) |
|  | After meals | 26 (9.2) |
|  | At social gatherings | 51 (18.0) |
|  | No specific time | 32 (11.3) |
| Location of e-cigarette use | |  |
|  | At home or in a dormitory | 33 (28.4) |
|  | At a friend's house or friend's dormitory | 4 (3.4) |
|  | In entertainment venues | 36 (31.0) |
|  | In educational institutions | 4 (3.4) |
|  | No specific location | 39 (33.6) |
| E-cigarette usage companions | |  |
|  | Close friends | 76 (35.2) |
|  | Alone | 67 (31.0) |
|  | Seniors/juniors | 36 (16.7) |
|  | Family | 4 (1.9) |
|  | Partner | 31 (14.4) |
|  | Others (friends, siblings) | 2 (0.9) |
| Source of e-cigarettes | |  |
|  | Purchased from online stores | 48 (30.2) |
|  | Via Line | 18 (11.3) |
|  | Via Facebook | 9 (5.7) |
|  | Asked someone else to buy it | 16 (10.1) |
|  | Bought second-hand from a friend or acquaintance | 2 (1.3) |
|  | Shared with friends | 11 (6.9) |
|  | Purchased from general stores | 53 (33.3) |
|  | Others | 2 (1.3) |
| Channels for e-cigarette advertising | |  |
|  | Social media and online marketplaces (e.g., Facebook, LINE, Shopee, Lazada) | 42 (36.2) |
|  | Friends | 25 (21.6) |
|  | Websites dedicated to selling e-cigarettes | 10 (8.6) |
|  | Direct transactions with sellers (no physical store/distributor) | 4 (3.4) |
|  | E-cigarette stores (with physical storefront)/street vendors/flea markets | 33 (28.4) |
|  | Others | 2 (1.7) |
| Trend in e-cigarette usage volume | |  |
|  | Decreased | 58 (50.0) |
|  | Remained the same | 58 (50.0) |
| Frequency of e-cigarette usage | |  |
|  | Decreased | 60 (51.7) |
|  | Remained the same | 56 (48.3) |
| Intention to quit e-cigarette use within the next month | |  |
|  | No | 32 (27.6) |
|  | Yes | 47 (40.5) |
|  | Unsure | 31 (31.9) |
| Intention to quit e-cigarette use within the next 6 months | |  |
|  | No | 35 (30.2) |
|  | Yes | 49 (40.2) |
|  | Unsure | 32 (27.6) |
| Attempts to quit e-cigarette use | |  |
|  | Never tried | 51 (44.0) |
|  | Tried | 29 (25.0) |
|  | Quit and relapsed | 36 (31.0) |

Note: Participants could select more than one response; therefore, percentages may not sum to 100%.

**Supplementary Table S4** Analysis of the number and percentage of respondents by ADHD symptom prevalence among e-cigarette users and non-smokers

| **ADHD Symptom Prevalence** | **Non-smoker (n= 116)**  **n (%)** | **E-cigarette user (n= 116)**  **n (%)** | **All participants (n= 232)**  **n (%)** |
| --- | --- | --- | --- |
| No symptoms consistent with ADHD | 104 (89.7) | 103 (88.8) | 207 (89.2) |
| Symptoms consistent with ADHD | 12 (10.3) | 13 (11.2) | 25 (10.8) |

Note: ADHD symptom tendency was assessed using the ASRS. A score ≥24 indicates ADHD-consistent symptoms.

**Supplementary Table S5** Descriptive statistics of EQ levels, including frequency, percentage (%), mean, and standard deviation (S.D.), categorized by "Good," "Competent," and "Well-Being" dimensions for all participants (n = 232)

| **Category** | **Below normal**  **n (%)** | **Normal**  **n (%)** | **Above normal**  **n (%)** | **Mean** | **S.D.** | **Results** |
| --- | --- | --- | --- | --- | --- | --- |
| **Good** | | | | | | |
| Self-regulation | 0 (0) | 129 (55.6) | 103 (4.4) | 2.44 | 0.50 | Normal |
| Empathy | 62 (26.7) | 128 (55.2) | 42 (18.1) | 1.91 | 0.66 | Below normal |
| Responsibility | 80 (34.5) | 137 (39.1) | 15 (6.5) | 1.72 | 0.58 | Below normal |
| Overall | 0 (0) | 125 (53.9) | 107 (46.1) | 2.02 | 0.57 | Normal |
| **Competent** | | | | | | |
| Motivation | 23 (9.9) | 180 (77.8) | 29 (12.5) | 2.03 | 0.47 | Normal |
| Decision-making | 31 (13.4) | 187 (80.6) | 14 (6.0) | 1.93 | 0.44 | Below normal |
| Relationship skills | 82 (35.3) | 129 (55.6) | 21 (9.1) | 1.74 | 0.61 | Below normal |
| Overall | 2 (0.9) | 189 (81.5) | 41 (17.7) | 1.91 | 0.50 | Below normal |
| **Well-being** | | | | | | |
| Self-pride | 9 (3.9) | 195 (84.1) | 28 (12.1) | 2.08 | 0.39 | Normal |
| Self-satisfaction | 76 (32.8) | 113 (48.7) | 43 (18.5) | 1.86 | 0.70 | Below normal |
| Inner peace | 36 (15.5) | 178 (76.7) | 18 (7.8) | 1.92 | 0.48 | Below normal |
| Overall | 2 (0.9) | 177 (76.3) | 53 (22.8) | 1.96 | 0.52 | Below normal |

**Note:** EQ = Emotional Quotient. Scores were classified as below normal (1.00–2.00), normal (2.01–3.00), and above normal (3.01–4.00). Values are presented as frequency (%).

**Supplementary Table S6** Descriptive statistics of EQ levels, including frequency, percentage (%), mean, and standard deviation (S.D.), categorized by "Good," "Competent," and "Well-Being" dimensions for non-smokers (n = 116)

| **Category** | **Non-smokers EQ levels (n=116)** | | | | | |
| --- | --- | --- | --- | --- | --- | --- |
|  | **Below normal**  **n (%)** | **Normal**  **n (%)** | **Above normal**  **n (%)** | **Mean** | **S.D.** | **Results** |
| **Good** | | | | | | |
| Self-regulation | 0 (0) | 61 (52.6) | 55 (47.4) | 2.47 | 0.50 | Normal |
| Empathy | 27 (23.3) | 70 (60.3) | 19 (16.4) | 1.93 | 0.63 | Below normal |
| Responsibility | 17 (14.7) | 88 (75.9) | 11 (9.5) | 1.72 | 0.50 | Below normal |
| Overall | 0 (0) | 57 (49.1) | 59 (50.9) | 2.04 | 0.54 | Normal |
| **Competent** | | | | | | |
| Motivation | 16 (13.8) | 92 (76.3) | 8 (6.9) | 1.93 | 0.45 | Below normal |
| Decision-making | 16 (13.8) | 94 (82.8) | 10 (6.9) | 1.90 | 0.40 | Below normal |
| Relationship skills | 40 (34.5) | 71 (61.2) | 5 (4.3) | 1.70 | 0.55 | Below normal |
| Overall | 2 (1.7) | 92 (79.3) | 22 (19.0) | 1.84 | 0.47 | Below normal |
| **Well-being** | | | | | | |
| Self-pride | 9 (7.8) | 102 (87.9) | 5 (4.3) | 1.97 | 0.35 | Below normal |
| Self-satisfaction | 32 (27.6) | 70 (60.3) | 14 (12.1) | 1.84 | 0.61 | Below normal |
| Inner peace | 17 (14.7) | 88 (75.9) | 11 (9.5) | 1.95 | 0.49 | Below normal |
| Overall | 2 (1.7) | 92 (79.3) | 22 (19.0) | 1.92 | 0.48 | Below normal |

**Note:** EQ = Emotional Quotient. Scores were classified as below normal (1.00–2.00), normal (2.01–3.00), and above normal (3.01–4.00). Values are presented as frequency (%).

**Supplementary Table S7** Descriptive statistics of EQ levels, including frequency, percentage (%), mean, and standard deviation (S.D.), categorized by "Good," "Competent," and "Well-Being" dimensions for e-cigarette users (n = 116)

| **Category** | **E-cigarette users EQ levels (n=116)** | | | | | |
| --- | --- | --- | --- | --- | --- | --- |
|  | **Below normal**  **n (%)** | **Normal**  **n (%)** | **Above normal**  **n (%)** | **Mean** | **S.D.** | **Results** |
| **Good** | | | | | | |
| Self-regulation | 0 (0) | 68 (58.6) | 48 (41.4) | 2.41 | 0.49 | Normal |
| Empathy | 35 (30.2) | 58 (50.0) | 23 (19.8) | 1.90 | 0.70 | Below normal |
| Responsibility | 40 (37.9) | 60 (51.7) | 12 (10.3) | 1.72 | 0.64 | Below normal |
| Overall | 0 (0) | 68 (58.6) | 48 (41.4) | 2.01 | 1.83 | Normal |
| **Competent** | | | | | | |
| Motivation | 7 (6.0) | 88 (75.9) | 21 (18.1) | 2.12 | 0.48 | Normal |
| Decision-making | 15 (12.9) | 91 (78.4) | 10 (8.6) | 1.96 | 0.46 | Below normal |
| Relationship skills | 42 (36.2) | 58 (50.0) | 16 (13.8) | 1.78 | 0.67 | Below normal |
| Overall | 0 (0) | 85 (73.3) | 31 (26.7) | 1.95 | 0.53 | Below normal |
| **Well-being** | | | | | | |
| Self-pride | 0 (0) | 93 (80.2) | 23 (19.8) | 2.20 | 0.40 | Normal |
| Self-satisfaction | 44 (37.9) | 43 (37.1) | 29 (25.0) | 1.87 | 0.77 | Below normal |
| Inner peace | 19 (16.4) | 90 (77.6) | 7 (6.0) | 1.90 | 0.46 | Below normal |
| Overall | 0 (0) | 85 (73.3) | 31 (26.7) | 1.99 | 0.54 | Below normal |

**Note:** EQ = Emotional Quotient. Scores were classified as below normal (1.00–2.00), normal (2.01–3.00), and above normal (3.01–4.00). Values are presented as frequency (%).
